# 836. Usability of an Artificial Intelligence-enhanced Multispectral Imaging Tool for Burn Assessment: A Retrospective Heuristic Evaluation

**DOI:** 10.1093/jbcr/irag033.275

**Published:** 2026-04-06

**Authors:** Hajar S Abdulla, Poh (Leslie) Tan, Zeeshan Sheikh, Christopher J Lewis

**Affiliations:** Royal Victoria Infirmary, Newcastle, Gosforth, Newcastle Upon Tyne, England; Royal Victoria Infirmary, Newcastle, Newcastle upon Tyne, England; Manchester NHS Foundation Trust, Manchester, England; Northern Regional Burn Centre, Newcastle Upon Tyne, England

## Abstract

**Introduction:**

Artificial intelligence (AI)–enhanced multispectral imaging (MSI) is an emerging technology for burn assessment. These devices offer objective predictions of burn healing potential however, their usability in clinical practice remains poorly understood. Key factors such as device handling, workflow integration, and interpretability are critical to successful adoption. This study evaluated the usability of an AI-enhanced MSI device, drawing on nursing and surgeon feedback mapped to consolidated usability heuristic domains.

**Methods:**

We conducted a multicentre retrospective evaluation across four Burn Centres using the device. Clinicians completed a structured survey comprising twelve Likert-scale statements (1 = strongly disagree, 5 = strongly agree), each mapped to six heuristic domains: visibility & recognition, match & standards, control & freedom, error prevention & recovery, flexibility & efficiency, and minimalism & aesthetics. Usability outcomes were reported as domain-specific means with standard deviations, medians with interquartile ranges, and proportions of positive responses (≥4). Between-group differences by clinical role were assessed using Mann–Whitney U tests, while site-level variability was examined with Kruskal–Wallis tests. Free-text responses were thematically analysed & mapped to heuristic domains to complement the quantitative findings.

**Results:**

Forty-seven clinicians responded (60% nurses, 40% surgeons). Overall usability ratings were high, with domain means ranging from 3.95 to 4.36. Flexibility and efficiency scored highest (mean 4.29; 87.9% positive) while match and standards scored lowest (mean 3.95; 65.9% positive). Surgeons rated match and standards slightly higher than nurses (mean 4.11 vs 3.86; 73.7% vs 60.7% positive), though this difference was not statistically significant (U = 302.0, *p*=.42). No statistically significant differences were found between hospitals across any domain (all *p*>.05). Free text comments emphasised ease of use & expressed interest in future refinements, including scanning hands & feet and providing more granular prognostic outputs.

**Conclusions:**

This study demonstrates that the AI-enhanced MSI device is both usable and acceptable across multidisciplinary burn teams, with consistent evaluations supporting its integration into routine practice. Its strengths in workflow efficiency & ease of handling highlight its potential as a practical adjunct to existing clinical assessment methods. Continued refinement & prospective validation will be essential to establish its role in optimising burn care delivery.

**Applicability of Research to Practice:**

These findings support the integration of AI enhanced MSI devices into burn care, with high usability across roles and sites, suggesting potential for broad adoption with minimal training. Targeted refinements could further streamline workflow and improve user experience.

**Funding for the study:**

N/A.

